# Negative coping and working in the NICU as key predictors of work fatigue risk among pediatric nurses: a cross-sectional study guided by the job demands-resources theory

**DOI:** 10.3389/fpubh.2026.1776586

**Published:** 2026-04-17

**Authors:** Pan Peng, Xiaolan Shu, Ying Jiang, Wenting Gong, Qin Li

**Affiliations:** Department of Pediatric Critical Care Medicine, Maternal and Child Health Hospital of Hubei Province, Wuhan, China

**Keywords:** factors, job demands-resources theory, nursing managers, pediatric nurse, work fatigue risk

## Abstract

**Objective:**

Guided by the Job Demands-Resources (JD-R) theory, this study aimed to reveal the current status of work fatigue risk among pediatric nurses and to identify the key influencing factors, in order to provide evidence for targeted interventions.

**Methods:**

A descriptive cross-sectional study was conducted. From February to March 2025, convenience sampling was used to select 325 pediatric nurses from a tertiary hospital in China as study participants. A questionnaire survey was conducted using the general information questionnaire, the Work Fatigue Risk Assessment Questionnaire for Nurses, Simplified Coping Style Scale, and Social Support Rating Scale. Univariate and multiple linear stepwise regression analyses were used to explore the influencing factors.

**Results:**

A total of 323 pediatric nurses were included in the final analysis, yielding a response rate of 99.4%. Their overall work fatigue risk score was 54.77 ± 12.78 (range: 22–110). Pediatric nurses scored highest on the personal state dimension (18.05 ± 4.52), followed by the organizational culture dimension (15.32 ± 4.66). For 323 pediatric nurses, work fatigue risk was negatively correlated with positive coping and social support, and positively correlated with negative coping (all *p* < 0.05). Multiple linear regression analysis revealed that sleep disorders (*β* = 0.281, *t* = 5.953, *p* < 0.001), working in the Neonatal Intensive Care Unit (NICU) (*β* = 0.320, *t* = 7.330, *p* < 0.001), negative coping (*β* = 0.333, *t* = 7.127, *p* < 0.001), positive coping (*β* = −0.241, *t* = −5.248, *p* < 0.001), and health status (*β* = 0.122, *t* = 2.621, *p* = 0.009) were independent risk factors of work fatigue among pediatric nurses, collectively explaining 39.3% of the total variance (adjusted *R*^2^ = 0.393, *F* = 42.647, *p* < 0.001).

**Conclusion:**

Pediatric nurses exhibited a moderate level of work fatigue risk. Guided by the JD-R theory, sleep disorders, working in the NICU, coping styles, and health status were key predictors of work fatigue risk. The findings provided a theoretical reference for nursing administrators to develop targeted, personalized interventions that reduce the work fatigue risk.

## Introduction

Work Fatigue is a work-related state characterized by extreme physical and mental exhaustion during or after work, accompanied by reduced work capacity (1). Studies (2, 3) have shown that fatigue among nurses contributes to adverse events such as medication errors, decreased nursing quality, and lower patient satisfaction. Prolonged work fatigue not only induces occupational burnout but also diminishes nurses’ sense of professional accomplishment (4). Pediatric nurses, in particular, experience high physical and mental stress due to prolonged interactions with ill children and their families (5). Additionally, the unique nursing context—including the acute onset of childhood illnesses and high hospital bed turnover—may further exacerbate their fatigue risk (6). Thus, it is recommended that nursing administrators conduct early, comprehensive assessments of work fatigue risk.

The Job Demands-Resources (JD-R) theory is a classic framework for explaining occupational stress and fatigue. This theory posits that every occupation has its unique job demands and job resources (7). Job demands refer to the mental and physical exertion required in the workplace and related factors. Job resources, on the other hand, encompass psychosocial and other factors that facilitate personal growth or the achievement of work goals. Per the JD-R theory, positive coping strategies and social support function as valuable resources in the workplace, playing a critical role in regulating work states and achieving work goals (7).

Existing research (8–10) on nurse fatigue has primarily focused on empathy fatigue among general clinical nurses, with study variables largely limited to demographic factors and basic job demands, such as age, years of working, and shift patterns. While these studies provide valuable insights, they rarely adopt a comprehensive theoretical framework that simultaneously examines both job demands and job resources. Moreover, pediatric nurses who face distinct workplace stressors including high-acuity patient conditions, emotionally charged family interactions, and fast-paced clinical environments, remain underrepresented in fatigue research (5, 6). Few studies have comprehensively assessed the level of work fatigue risk among pediatric nurses from both individual and organizational perspectives. Consequently, there is a notable gap in understanding how job demands (e.g., department, health status) and job resources (e.g., coping styles, social support) jointly influence work fatigue risk in this population, particularly within the integrative structure of the JD-R theory.

To address this gap, this study explored the current status of work fatigue risk among pediatric nurses from both individual and organizational perspectives and applied the JD-R theory as an overarching framework to identify the key influencing factors of work fatigue risk among pediatric nurses. Guided by the JD-R theory, we operationalized job demands as factors that require sustained physical or psychological effort from nurses, including: department (with NICU and PICU representing high-demand settings), health status, education level, sleep disorders, years of working, technical title, position, specialized nursing certification, average weekly night shifts. Job resources were operationalized as factors that facilitate coping, goal achievement, or buffer against job demands, including: positive coping style, negative coping style, and social support. Specifically, this study addressed the following research questions (RQs):

RQ1: What is the current status of work fatigue risk among pediatric nurses?

RQ2: How do job demands and job resources predict work fatigue risk guided by the JD-R theory?

The findings aimed to provide a theoretical reference for nursing administrators to develop targeted, personalized interventions that reduce work fatigue risk.

## Methods

### Design

This study adopted a descriptive cross-sectional design to investigate the current prevalence of work fatigue risk and its associated factors among pediatric nurses at a single time point. This design effectively addresses practical constraints commonly encountered in longitudinal research, such as high nurse turnover, thereby achieving a balance between research feasibility and data validity. As an initial exploratory investigation, it provides empirical baseline evidence to support future longitudinal and intervention studies. Moreover, considering the heavy workload of pediatric nurses, the cross-sectional survey approach minimizes the respondent burden while ensuring a sufficiently representative sample for reliable statistical analysis.

### Participants and recruitment

Convenience sampling was employed to recruit pediatric nurses from a tertiary grade A hospital in China between February and March 2025. Inclusion criteria: (1) Currently employed as a registered pediatric nurse; (2) At least 1 year of clinical experience in pediatric nursing; (3) Obtained informed consent and voluntarily participated in this survey. Exclusion criteria: (1) Nurses not on duty during the survey period (e.g., on leave, sick leave, or external training); (2) In the lactation period (lactation induces hormonal fluctuations and sleep fragmentation that independently alter fatigue levels (11), and lactating nurses in China are entitled to statutorily mandated workplace accommodations, which result in non-standard job demands and resources); (3) Not formally employed by the hospital (e.g., visiting nurses). This study involved 16 independent variables. Based on the empirical rule that the sample size should be 10 to 20 times the number of independent variables (12), and taking into account a potential non-response rate of 10% in the actual survey, the theoretically required sample size was 178 to 356.

### Data collection

Data were collected using the Wenjuanxing online platform, a widely used and secure professional online survey platform in China. This online platform was used to distribute a single integrated questionnaire consisting of four components: a general information questionnaire, the Work Fatigue Risk Assessment Questionnaire for Nurses, the Simple Coping Style Scale, and the Social Support Rating Scale. Participants completed all four components sequentially within this single survey instrument. Following approval from the head nurses of each pediatric department, questionnaire links or QR codes were distributed to eligible nurses. Participants completed the questionnaire anonymously, with mandatory completion of all items before submission. The preface of the questionnaire provided detailed information on the completion guidelines to ensure data quality. A total of 325 questionnaires were distributed. Preliminary pilot study data showed that at least 2 min was needed to carefully read and complete all questionnaire items, and responses finished in under 2 min were considered as inattentive or random completion.

### Data collection tools

#### General information questionnaire

Developed by the research team based on a literature review and data collection, this questionnaire included 13 items: department, gender, age, marital status, health status, number of children, education level, sleep disorders, years of working, technical title, position, specialized nursing certification, average weekly night shifts.

#### Work fatigue risk assessment questionnaire for nurses

Developed by Pi et al. (13) in 2025, this questionnaire was based on the “2–4” model framework of accident causation. The questionnaire consists of four dimensions—personal competence, personal state, organizational culture, and management system, with 22 items in total. Each item uses a 5-point Likert scale (1 = “Never” to 5 = “Always”). Total scores range from 22 to 110, with higher scores indicating higher work fatigue risk. It can be used as a reliable tool for assessing the work fatigue risk in clinical nurses. The total Cronbach’s *α* coefficient of original validation study was 0.962.

#### Simple Coping Style Scale

This scale comprises two dimensions: positive coping (items 1–12) and negative coping (items 13–20) (14). A 4-point Likert scale is used (0 = “Never adopted” to 3 = “Frequently adopted”). Total scores of positive coping range from 0 to 36, and total scores of negative coping range from 0 to 24, with higher scores indicating a stronger tendency to use that coping style. It reflects the individual coping styles. The overall Cronbach’s *α* coefficient of original validation study was 0.90, while the Cronbach’s α coefficients for the positive coping and negative coping dimensions were 0.89 and 0.78, respectively.

#### Social Support Rating Scale

This scale includes 10 items across three dimensions: objective support, subjective support, and utilization of social support (15). Item 5 is calculated as the sum of four sub-items (A–D), each rated on a 4-point Likert scale ranging from 1 (“None”) to 4 (“Full support”). For items 6 and 7, a score of 0 is assigned if “No source” is selected; otherwise, the score corresponds to the number of sources selected. All remaining items are rated on a 4-point Likert scale from 1 to 4. Total scores range from 12 to 66, with higher scores indicating greater social support. It was used to evaluate the level of social support. The total Cronbach’s *α* coefficient of original validation study was 0.92.

### Psychometric properties of data collection tools

The validity of the three main scales (Work Fatigue Risk Assessment Questionnaire, Simple Coping Style Scale, and Social Support Rating Scale) was established in their original developmental studies (13–15), which confirmed their construct and content validity. Given their widespread use and robust psychometric evidence in nursing research, this study adopted these scales directly without re-conducting factor analysis for construct validity.

However, to ensure the instruments’ applicability to our specific sample, we assessed reliability using Cronbach’s *α* coefficient based on the data from the 323 participants. Additionally, content validity was ensured through a pilot study and expert review prior to formal data collection. The results indicated that the overall Cronbach’s *α* coefficient for the Work Fatigue Risk Assessment Questionnaire was 0.903. The overall Cronbach’s *α* coefficient of the Simple Coping Style Scale was 0.884, while the Cronbach’s *α* coefficients for the positive coping and negative coping dimensions were 0.918 and 0.792, respectively. The Cronbach’s *α* coefficient of the Social Support Rating Scale was 0.787. These values exceed the commonly accepted threshold of 0.70, confirming the internal consistency of the instruments for the current sample.

### Data analysis

Statistical analysis was performed using SPSS 27.0 software. Continuous variables with a normal or approximately normal distribution were presented as Mean ± standard deviation (SD). Independent samples *t*-tests or one-way ANOVA were used to compare differences in the scores of work fatigue risk among pediatric nurses across different characteristics. Pearson correlation analysis was conducted to examine the relationships between work fatigue risk, coping styles, and social support. Multiple linear regression (stepwise method) was used to identify independent influencing factors of work fatigue risk. *p* < 0.05 was considered statistically significant difference.

### Ethical considerations

This study was approved by the Ethics Committee of the Maternal and Child Health Hospital of Hubei Province (Approval Number: 2025-072-01; Approval Date: May 9, 2025). All participants provided informed consent and voluntarily participated in this study. The preface of the questionnaire provided detailed information on the study’s purpose and content to ensure written informed consent.

## Results

### Work fatigue risk, coping styles and social support among pediatric nurses

After excluding questionnaires with obvious errors or those completed in less than 2 min, a total of 323 pediatric nurses were included in the final analysis, yielding a valid response rate of 99.4%. For these pediatric nurses, their overall work fatigue risk score was 54.77 ± 12.78, ranging from 22 to 110. Among the four dimensions of work fatigue risk, pediatric nurses scored highest on the personal state dimension (18.05 ± 4.52), followed by the organizational culture dimension (15.32 ± 4.66). In terms of coping styles, pediatric nurses reported a mean positive coping score of 22.42 ± 6.70 (range: 0–36) and a mean negative coping score of 10.48 ± 4.40 (range: 0–24). Regarding social support, pediatric nurses had an average score of 37.97 ± 8.31, with individual scores ranging from 12 to 66 (Table 1).

**Table 1:** Total and dimension scores of work fatigue risk, coping styles, and social support in pediatric nurses.

Item	Number of items	Total score (mean ± SD)	Score per item (mean ± SD)
Work fatigue risk	22	54.77 ± 12.78	2.49 ± 0.58
Personal competence dimension	6	11.66 ± 3.68	1.94 ± 0.61
Personal state dimension	6	18.05 ± 4.52	3.01 ± 0.75
Organizational culture dimension	6	15.32 ± 4.66	2.55 ± 0.78
Management system dimension	4	9.75 ± 3.67	2.44 ± 0.92
Coping style			
Positive coping dimension	12	22.42 ± 6.70	1.87 ± 0.56
Negative coping dimension	8	10.48 ± 4.40	1.31 ± 0.55
Social support	10	37.97 ± 8.31	3.80 ± 0.83

### Univariate differences in work fatigue risk among pediatric nurses with distinct characteristics

Pediatric nurses exhibited statistically significant differences in work fatigue risk across different departments, health status, sleep disorders, years of working, and average weekly night shifts (all *p* < 0.01), as detailed in Table 2.

**Table 2:** Univariate analysis of work fatigue risk among pediatric nurses.

Variables	Category	*N* (%)	Score (mean ± SD)	Value of the test statistic	*p*-value
Department	Pediatrics department	109 (33.7)	51.90 ± 11.54	8.982^2^	<0.001
	Department of pediatric surgery	41 (12.7)	52.34 ± 13.77		
	Pediatrics outpatient department	22 (6.8)	49.41 ± 10.74		
	Pediatric emergency department	49 (15.2)	53.29 ± 13.23		
	Pediatric intensive care unit (PICU)	38 (11.8)	56.45 ± 9.17		
	Neonatal intensive care unit (NICU)	64 (19.8)	63.19 ± 12.69		
Gender	Male	6 (1.9)	53.67 ± 14.29	−0.213^1^	0.832
	Female	317 (98.1)	54.79 ± 12.77		
Age	≤25	34 (10.5)	53.00 ± 13.44	1.923^2^	0.148
	26~35	175 (54.2)	53.91 ± 13.30		
	≥36	114 (35.3)	56.61 ± 11.60		
Marital status	Unmarried	73 (22.6)	53.01 ± 13.27	−1.335^1^	0.183
	Married/divorced/widowed	250 (77.4)	55.28 ± 12.61		
Health status	Good	176 (54.5)	51.35 ± 12.21	15.515^2^	<0.001
	Fair	137 (42.4)	58.62 ± 12.46		
	Poor	10 (3.1)	62.20 ± 8.89		
Number of children	0	104 (32.2)	52.78 ± 13.73	2.032^2^	0.133
	1	152 (47.1)	55.39 ± 12.57		
	≥2	67 (20.7)	56.45 ± 11.43		
Education level	Associate degree or below	47 (14.6)	54.23 ± 11.74	−0.309^1^	0.757
	Bachelor’s degree or above	276 (85.4)	54.86 ± 12.96		
Sleep disorders	No	136 (42.1)	48.50 ± 11.81	−8.269^1^	<0.001
	Yes	187 (57.9)	59.33 ± 11.48		
Years of working	≤10	141 (43.7)	52.20 ± 13.35	5.194^2^	0.006
	11~20	164 (50.8)	56.78 ± 12.18		
	≥21	18 (5.6)	56.56 ± 10.15		
Technical title	Nurse	34 (10.5)	53.29 ± 12.58	0.939^2^	0.392
	Practicing nurse	185 (57.3)	54.28 ± 13.24		
	Senior nurse or above	104 (32.2)	56.12 ± 11.98		
Position	None	313 (96.9)	54.75 ± 12.87	−0.159^1^	0.874
	Head nurse	10 (3.1)	55.40 ± 9.79		
Specialized nursing certification	No	271 (83.9)	54.59 ± 13.12	−0.557^1^	0.578
	Yes	52 (16.1)	55.67 ± 10.85		
Average weekly night shifts	0	42 (13.0)	48.88 ± 10.90	15.311^2^	<0.001
	1	223 (69.0)	54.00 ± 12.75		
	≥2	58 (18.0)	61.97 ± 11.11		

1 *t*-value.

2 *F*-value.

### Correlations between work fatigue risk, coping styles and social support in pediatric nurses

Pearson correlation analysis revealed that for pediatric nurses, work fatigue risk was weakly negatively correlated with positive coping (*r* = −0.162, *p* < 0.05) and social support (*r* = −0.196, *p* < 0.001), while moderately and positively correlated with negative coping (*r* = 0.314, *p* < 0.001). Specifically, regarding the sub-dimensions of work fatigue risk, pediatric nurses’ personal competence and personal state scores were both negatively correlated with positive coping and social support, and positively correlated with negative coping (all *p* < 0.05). Their scores on the organizational culture dimension showed a weak positive correlation with negative coping (*r* = 0.285, *p* < 0.001). Similarly, the management system dimension was weakly positively associated with negative coping (*r* = 0.249, *p* < 0.001) and negatively associated with social support (*r* = −0.110, *p* < 0.05) (Table 3).

**Table 3:** Correlation analysis of work fatigue risk with coping styles and social support in pediatric nurses.

Variables	Work fatigue risk	Personal competence	Personal state	Organizational culture	Management system
Coping style					
Positive coping	−0.162^2^	−0.213^1^	−0.160^2^	−0.085	−0.044
Negative coping	0.314^1^	0.216^1^	0.216^1^	0.285^1^	0.249^1^
Social support	−0.196^1^	−0.237^1^	−0.185^1^	−0.084	−0.110^2^

1 *p* < 0.001.

2 *p* < 0.05.

### Multivariate linear regression analysis of work fatigue risk among pediatric nurses

Taking the total work fatigue risk score of pediatric nurses as the dependent variable and statistically significant variables from univariate and correlation analyses as independent variables, stepwise multiple linear regression was performed. Before performing the regression analysis, the tolerance and Variance Inflation Factor (VIF) values were calculated to test for multicollinearity. The VIF values for all variables were less than 5, indicating no significant multicollinearity between the independent variables. The coding methods for the independent variables were specified as follows: Department: Pediatrics Department (0,0,0,0,0); Department of Pediatric Surgery (1,0,0,0,0); Pediatrics Outpatient Department (0,1,0,0,0); Pediatric Emergency Department (0,0,1,0,0); PICU (0,0,0,1,0); NICU (0,0,0,0,1). Health status: Good = 1; Fair = 2; Poor = 3. Sleep disorder: No = 0; Yes = 1. Years of working: ≤10 = 1; 11~20 = 2; ≥21 = 3. Average weekly night shifts: 0 = 1; 1 = 2; ≥2 = 3. The scores of positive coping, negative coping, and social support were all entered as raw values. The results showed that sleep disorders, working in the NICU, negative coping, positive coping, and health status were independent influencing factors of work fatigue risk, explaining 39.3% of the total variance (adjusted *R*^2^ = 0.393, *F* = 42.647, *p* < 0.001). Among them, negative coping (*β* = 0.333, *p* < 0.001) had the strongest positive impact on the work fatigue risk and was the most significant risk factor. Working in the NICU (*β* = 0.320, *p* < 0.001) was the second strongest factor with a positive predictive effect. Conversely, positive coping (*β* = −0.241, *p* < 0.001) was significantly associated with a reduced risk of work fatigue, indicating a moderate protective effect. (Table 4).

**Table 4:** Multivariate linear regression analysis of work fatigue risk among pediatric nurses.

Variables	Unstandardized coefficients	Standard error	Standardized coefficients	*t*-value	*p*-value
Constant	44.585	2.551		17.475	<0.001
Sleep disorders	7.254	1.219	0.281	5.953	<0.001
Working in the NICU	10.245	1.398	0.320	7.330	<0.001
Negative coping	0.965	0.135	0.333	7.127	<0.001
Positive coping	−0.460	0.088	−0.241	−5.248	<0.001
Health status	2.790	1.064	0.122	2.621	0.009

*R*^2^ = 0.402, adjusted *R*^2^ = 0.393, *F* = 42.647, *p* < 0.001.

## Discussion

This study found that the total score of work fatigue risk among pediatric nurses was 54.77 ± 12.78, indicating a moderate level of risk. This finding is consistent with the results of Arikan and Esenay (16) among Turkish pediatric emergency nurses during the COVID-19 pandemic, who reported moderate compassion fatigue levels, Besides, it aligns with recent studies from Iran (17) and the United States (5) indicating that pediatric nurses experience substantial occupational fatigue. However, the risk level observed in our sample was slightly higher than the empathy fatigue level reported in general clinical nurses by Ahmadi et al. (17) in Iran. The discrepancy may be attributed to the unique challenges of pediatric nursing: pediatric nurses must manage rapid changes in children’s conditions and meet high family expectations for nursing quality. In critical scenarios (e.g., rescuing critically ill children), these job demands are associated with increased work fatigue risk (18). Among the four dimensions of work fatigue risk, the personal state dimension had the highest score, followed by the organizational culture dimension. It reflected that individual factors, such as irregular eating habits and inadequate rest, are primary drivers linked to work fatigue risk. Additionally, organizational factors (e.g., lack of free psychological counseling and noisy rest environments) contribute to work fatigue risk, highlighting the dual-level nature of work fatigue as conceptualized by the JD-R theory.

Guided by the JD-R theory, multiple linear regression analysis revealed that pediatric nurses with sleep disorders were associated with a higher work fatigue risk. This result was consistent with findings from international studies. Loew et al. (19) demonstrated that sleep disturbances among pediatric medical providers during night shifts negatively impacted clinical performance and well-being. Similarly, a systematic review by Zhang et al. (20) across multiple countries confirmed that poor sleep quality is a consistent predictor of occupational fatigue among nurses globally. Prolonged sleep deprivation or poor sleep quality is associated with impaired attention and judgment, which may require nurses to expend more effort to maintain job demands and care quality, potentially increasing fatigue and reducing quality of life (20). Moreover, pediatric nurses with sleep disorders often experience depleted psychological resilience due to work-related stress, making it difficult to regulate emotions. Over time, this may exacerbate fatigue risk (21, 22). Therefore, guided by the JD-R theory, sleep disorders can be understood as a job demand that depletes personal energy reserves, thereby exacerbating work fatigue risk.

This study indicated that nurses working in the NICU were associated with a higher risk of work fatigue compared to nurses working in Pediatrics Department. This finding is supported by international evidence: Barr (23) in Australia reported elevated stress levels among NICU nurses associated with high emotional demands, and Hagen et al. (24) recently confirmed high rates of compassion fatigue in NICU settings across multiple European centers. This may be due to the unique job demands of NICU patients: newborns have fragile physiological functions and poor environmental adaptability, requiring nurses to assume greater responsibility and face higher work pressure (23). In JD-R terms, the NICU environment represents a context characterized by high job demands (e.g., continuous monitoring, end-of-life care, complex technical procedures) and often limited job resources (e.g., insufficient psychological counseling, inadequate staffing). The imbalance between high job demands and limited job resources frequently leads NICU nurses to overlook their own fatigue while managing tasks (25). With advances in critical care medicine and inter-hospital transports (26), NICU patients are increasingly premature or critically ill, and are required high-precision, continuous care and extensive family communication (e.g., disease explanation, health guidance). These demands are further linked to an elevated fatigue risk (24).

An important theoretical contribution of this study lay in discovering the prominent role that coping styles played as personal job resources. Our results showed that negative coping was the strongest predictor of work fatigue risk. Moreover, the findings revealed that pediatric nurses with higher scores on negative coping styles, that is, those more inclined to avoid or accept reality when encountering difficulties or problems, were associated with higher levels of work fatigue risk. Conversely, those who favored positive coping strategies might have lower work fatigue risk. This finding aligned with the research by Zhou et al. (27) in China and Kubo et al. (28) in Japan, both of which identified coping styles as critical determinants of health outcomes among nurses. Coping styles, as a personal job resource, reflect an individual’s attitudes and abilities in dealing with stress (28). Studies (29) have shown that positive coping promotes optimism and healthy lifestyles, effectively alleviating work pressure. In contrast, negative coping (e.g., avoiding problems or self-blaming) as an ineffective personal job resource, is associated with internal conflict and may increase work fatigue risk over time. Within the JD-R framework, these findings reflected that job resources, particularly personal coping resources, can buffer the adverse effects of job demands on fatigue outcomes.

In this study, pediatric nurses with poor health status were associated with the higher level of work fatigue risk, consistent with findings by Zhong et al. (30) in China. A study on work fatigue and sickness absence found that acute fatigue directly impairs pediatric nurses’ physical health, increasing absenteeism (31). Good health helps nurses maintain work efficiency, thereby reducing fatigue and absenteeism. A study on ICU nurses also showed that physical health status was associated with presenteeism, which negatively impacts nursing quality (32). As a result, from the JD-R theory perspective, poor health status can be conceptualized as both a consequence of chronic job demands that amplifies the impact of ongoing demands on fatigue risk.

Guided by the JD-R theory, this study identified the key factors affecting the work fatigue risk among pediatric nurses, enriched the application of JD-R theory in the field of fatigue in pediatric nurses. Meanwhile, it provided a theoretical reference for managers to identify high-risk groups and reduce the work fatigue risk. This study has several limitations: First, it used a single-center, convenience sample, which may limit the generalizability of the findings. In future studies, a multi-center, stratified random sampling and large-sample design should be adopted to improve generalizability. Second, it focused on quantitative data, lacking qualitative insights into nurses’ subjective experiences of work fatigue risk. Integrating qualitative and quantitative methods will help explore fatigue risk-related factors more comprehensively in future studies. Third, this study only used correlation and multiple linear regression to screen key predictors. Although stepwise regression helped us identify the most significant factors, it has limitations in reflecting the complex mediating relationships between variables. Based on the JD-R theory, future research will incorporate a broader range of variables and systematically utilize structural equation modeling (SEM) and mediation analysis to verify the causal relationships between job demands, job resources, and work fatigue risk. Additionally, longitudinal studies are needed to examine the dynamic relationships between influencing factors and work fatigue risk, providing a more robust theoretical basis for evidence-based interventions.

## Conclusion

This study surveyed 323 pediatric nurses and found that the level of work fatigue risk was at a moderate level. Guided by the JD-R theory, this study explored that sleep disorders, working in the NICU, coping styles, and health status were key predictors of work fatigue risk. The findings provided a theoretical reference for nursing administrators to develop targeted, personalized interventions that reduce the work fatigue risk. Besides, future research can utilize structural equation modeling to explore potential mediating variables proposed by the JD-R theory.

### Implications for clinical practice

Clinical nursing managers should prioritize the work fatigue risk among pediatric nurses and focus on identifying high-risk groups. In particular, early intervention should be implemented for nurses in NICU and those with sleep disorders or poor health. Given that negative coping styles are the strongest predictor of work fatigue risk, hospitals should establish systematic psychological support mechanisms to guide nurses toward adopting positive coping styles rather than avoidance. These targeted measures will enhance the professional well-being of nurses.

## Data Availability

The raw data supporting the conclusions of this article will be made available by the authors, without undue reservation.
